# Association between Serum Uric Acid Levels and Diabetes Mellitus

**DOI:** 10.1155/2011/604715

**Published:** 2011-11-02

**Authors:** Pavani Bandaru, Anoop Shankar

**Affiliations:** ^1^Department of Community Medicine, West Virginia University School of Medicine, Morgantown, WV 26506, USA; ^2^Department of Medicine, West Virginia University School of Medicine, P.O. Box 9190, Morgantown, WV 26506-9190, USA

## Abstract

Serum uric acid has been shown to be associated with cardiovascular disease, hypertension, and chronic kidney disease in previous studies. However, few studies have examined the association between serum uric acid and diabetes mellitus and their findings are not consistent. Therefore, we examined the association between serum uric acid levels and diabetes mellitus in participants from the third National Health and Nutrition Examination Survey (*n* = 18, 825, 52.5% women). Serum uric acid levels were categorized into quartiles. Diabetes mellitus was defined as fasting glucose ≥126 mg/dL, nonfasting glucose ≥200 mg/dL, or use of oral hypoglycemic medication or insulin (*n* = 395). In multivariable logistic regression models, we found that higher serum uric acid levels were inversely associated with diabetes mellitus after adjusting for age, sex, race/ethnicity, education, smoking, alcohol intake, body mass index, hypertension, and serum cholesterol. Compared to quartile 1 of serum uric acid, the odds ratio (95% confidence interval) of diabetes mellitus was 0.48 (0.35–0.66; *P* trend <0.0001). The results were consistent in subgroup analysis by gender and hypertension status. Higher serum uric acid levels were inversely associated with diabetes mellitus in a representative sample of US adults.

## 1. Introduction

Serum uric acid, an end product of purine metabolism, has been shown to be associated with an increased risk of hypertension [1–3], cardiovascular disease [2, 4], and chronic kidney disease [5] in previous epidemiological studies. Also, elevated levels of uric acid is a risk factor for peripheral arterial disease [6], insulin resistance, and components of the metabolic syndrome [7]. However, the putative association between serum uric acid levels and diabetes mellitus is not clear. Some studies reported that there is a positive association between high serum uric acid levels and diabetes [8–13], whereas other studies reported no association [14], or an inverse relationship [15, 16]. In this context, the main purpose of our study was to examine the association between serum uric acid and prevalent diabetes in a large nationally representative sample of US adults after adjusting for major confounders. We had adequate sample size to examine this association in the whole cohort as well as separately by gender and hypertension.

## 2. Methods

The current study is based on data from the third National Health and Nutrition Examination Survey (NHANES III). Detailed description of NHANES III study design and methods are available elsewhere [17–20]. In brief, the NHANES survey included a stratified multistage probability sample representative of the civilian noninstitutionalized US population. Selection was based on counties, blocks, households, and individuals within households, and it included the oversampling of non-Hispanic blacks and Mexican Americans in order to provide stable estimates of these groups. Subjects were required to sign a consent form before their participation, and approval was obtained from the Human Subjects Committee in the US Department of Health and Human Service.

The current study sample consisted of participants aged greater than 20 years who were randomly assigned to be examined in the morning exam after an overnight fast. Serum uric acid levels were measured in 17,008 participants who were examined in the morning after an overnight fast who had surplus sera available. We further excluded subjects with self-reported cardiovascular disease (*n* = 1,521) and also subjects with missing data (*n* = 1,343) on covariates included in the multivariable model, including systolic or diastolic blood pressure, body mass index (BMI), or cholesterol levels. This resulted in 14,144 participants (52.5% women), 1,021 of whom had diabetes mellitus. 

## 3. Main Outcome of Interest

Serum glucose was measured using the modified hexokinase method at the University of Missouri, Diabetes Diagnostic Laboratory. Diabetes was defined based on the guidelines of the American Diabetes Association as a serum glucose ≥126 mg/dL after fasting for a minimum of 8 hours, a serum glucose ≥200 mg/dL for those who fasted <8 hours before their NHANES visit, or a self-reported current use of oral hypoglycemic medication or insulin.

## 4. Exposure Measurements 

Age, gender, race/ethnicity, smoking status, alcohol intake (g/day), level of education, history of diabetes and oral hypoglycemic intake or insulin administration, and antihypertensive medication use were assessed using a questionnaire. Individuals who had not smoked ≥100 cigarettes in their lifetimes were considered never smokers; those who had smoked ≥100 cigarettes in their lifetimes were considered former smokers if they answered negatively to the question “Do you smoke now?” and current smokers if they answered affirmatively. Body mass index (BMI) was calculated as weight in kilograms divided by height in meters squared. Serum total cholesterol was measured enzymatically.

Rigorous procedures with quality control checks were used in blood collection, and details about these procedures are provided in the NHANES Laboratory/Medical Technologists Procedures Manual [18–20]. Measurement of serum uric acid was performed by Collaborative Laboratory Services at Ottumwa, Iowa, using Beckman Synchron LX20 method. The LX20 uses a timed endpoint method to measure the concentration of uric acid in serum. Uric acid is oxidized by uricase to produce allantoin and hydrogen peroxide. The hydrogen peroxide reacts with 4-aminoantipyrine (4-AAP) and 3,5-dichloro-2-hydroxybenzene sulfonate (DCHBS) in a reaction catalyzed by peroxidase to produce a colored product. The system monitors the change in absorbance at 520 nm at a fixed time interval. The change in absorbance is directly proportional to the concentration of uric acid in the sample. 

## 5. Statistical Analysis

Serum uric acid was analyzed as a categorical variable. We categorized serum uric acid level as quartiles (<4.3 mg/dL, 4.30–5.20 mg/dL, 5.30–6.20 mg/dL, >6.20 mg/dL). 

The odds ratio [(OR) (95% confidence interval (CI)] of diabetes mellitus for each higher uric acid quartile was calculated by taking the lowest quartile as the referent, using multivariable logistic regression models. We used two models: the age- and sex-adjusted model, and the multivariable model, additionally adjusting for race/ethnicity (non-Hispanic whites, non-Hispanic blacks, Mexican Americans, etc.), education categories (below high school, high school, above high school), smoking (never smoker, former smoker, current smoker), alcohol intake (g/day), BMI (normal, overweight, obese), hypertension (absent, present), and total serum cholesterol (mg/dL). Trends in the OR of diabetes mellitus across increasing serum uric acid category were determined by modeling uric acid categories as an ordinal variable. Sample weights that account for the unequal probabilities of selection, oversampling, and nonresponse were applied for all analyses using SUDAAN (version 8.0; Research Triangle Institute, Research Triangle Park, NC, USA) and SAS (version 9.2; SAS Institute, Cary, NC, USA) software; SEs were estimated using the Taylor series linearization method. To examine the dose-response relationship between the observed association between serum uric acid levels and diabetes mellitus without linearity assumptions, we used flexible nonparametric logistic regression employing the generalized additive modeling approach (R system for statistical computing, available from Comprehensive R Archive Network (http://www.CRAN.R-project.org/)) and calculated the odds of diabetes mellitus, adjusting for all covariates in the multivariable model. The odds of diabetes mellitus were then plotted against increasing serum uric acid levels (both on the log scale) [21]. 

## 6. Results

Table 1 presents the baseline characteristics of the study population by increasing quartiles of serum uric acid level. Individuals in the higher uric acid quartiles were more likely to be older, non-Hispanic blacks, overweight and obese and have high total cholesterol levels.

Table 2 presents the association between increasing serum uric acid levels and diabetes mellitus in the whole cohort. We observed an inverse association between serum uric acid levels and diabetes mellitus in both the age- sex-adjusted and the multivariable-adjusted models. In a supplementary analysis where we examined the association between uric acid and diabetes mellitus defined in addition to fasting glucose as raised HbA1C (levels > 6.5%), compared to quartile 1 (referent) the multivariable adjusted odds ratio (95% CI) of diabetes in quartile 2 was 0.61 (0.42–0.89), quartile 3 was 0.50 (0.38–0.65), and in quartile 4 was 0.61 (0.45–0.83); *P* trend = 0.004.

**Figure 1:**
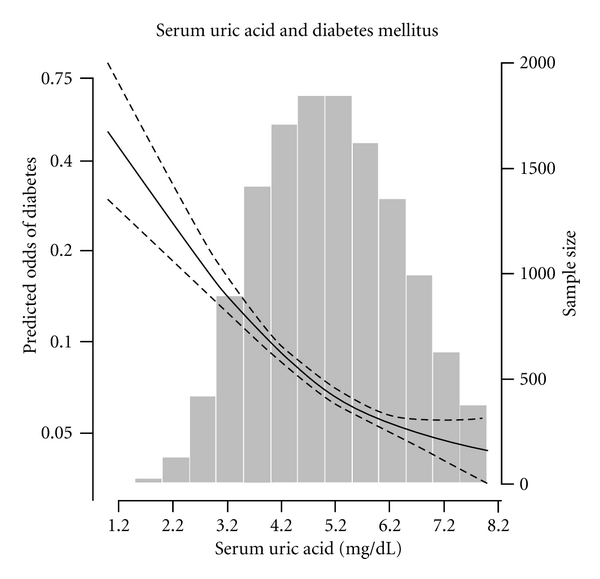
Multivariable-adjusted odds of diabetes mellitus according to serum uric acid level. Solid thick line represents the predicted odds of diabetes estimated from nonparametric logistic regression employing the generalized addictive-modeling approach (R system for statistical computing, Comprehensive R Archive Network (http://www.CRAN.R-project.org/)); dashed lines, 95% confidence limits for the nonparametric logistic regression estimates. The nonparametric logistic regression was adjusted for age (years), sex (men, women), race-ethnicity (non-Hispanic whites, non-Hispanic blacks, Mexican Americans, etc.), education categories (<high school, high school, >high school), smoking (never, former, current), alcohol intake (never, former, current), BMI (normal, overweight, obese), and serum total cholesterol (mg/dL). *x* axis: serum uric acid level (mg/dL) plotted in log scale, *y*1 axis: predicted odds of diabetes mellitus plotted in log scale, and *y*2 axis: participant number for each serum uric acid level.

In Table 3, the inverse association between increasing serum uric acid levels and diabetes mellitus was consistently present among men and women; however, the association was stronger in men.  Table 4 presents the association between increasing serum uric acid levels and diabetes mellitus by hypertension status. Consistent with the findings in Table 2 there was an inverse relationship between increasing serum uric acid levels and diabetes mellitus in nonhypertensive subjects and hypertensive subjects in multivariable-adjusted model. We then employed nonparametric models to examine if the observed inverse association between serum uric acid and diabetes mellitus was present across the full range of serum uric acid levels available in the present study (Figure 1). Overall, there appeared to be a continuous inverse association between serum uric acid and diabetes mellitus with increasing serum uric acid levels; there is no evidence of any threshold effect. 

## 7. Discussion

Higher serum uric acid levels were found to be inversely associated with diabetes mellitus in a large, contemporary, multiethnic sample of US adults. The observed inverse relation between serum uric acid and diabetes mellitus appeared to be independent of potential confounders and was consistently present in subgroup analyses by gender and hypertension. In a subsequent analysis, employing nonparametric models, the observed inverse association between serum uric acid quartiles and diabetes mellitus was present continuously across the full range of serum uric acid.

Previous studies examining the association between serum uric acid levels and diabetes mellitus were restricted to specific racial/ethnic groups and gender and were not consistent in their findings. Some studies reported that there is a positive association between elevated serum uric acid levels and diabetes [8–13], whereas some other study reported no positive association between serum uric acid and diabetes mellitus [14]. Also, some studies reported that serum uric acid is inversely associated with diabetes mellitus [15, 16]. The exact reason for why previous studies found a positive relation between uric acid and diabetes is not clear. Most of these studies were limited by small sample sizes, including either men or women and not both, not having data on confounding factors, or were from selected populations such as industrial workers as opposed to general population samples.

A plausible mechanism for the observed results of an inverse association between increasing serum uric acid and diabetes mellitus may be related to the inhibition of uric acid reabsorption in the proximal tubule by high glucose levels in diabetic individuals [22, 23]. To clarify the independent relation between serum uric acid levels and diabetes mellitus, we decided to adjust for all potential confounders and do subgroup analysis by gender. We found that in the current study, an inverse association was observed between elevated serum uric acid and diabetes mellitus even after adjusting for age, sex, race-ethnicity, education, smoking, alcohol intake, BMI, hypertension, and serum total cholesterol in both subgroup analysis by gender and hypertension. The large sample size available for the current analyses, our ability to adjust for all potential confounders, and the consistency of these results after subgroup analysis by gender and hypertension suggests that our findings are less likely due to chance. Therefore, higher uric acid levels may not be a risk factor for diabetes mellitus as some researchers previously argued [8–13]. 

The main strengths of our study include its population-based nature, inclusion of a representative multiethnic sample, and the availability of data on confounders for multivariable adjustment. We had a large sample size that enabled us to perform separate analysis by gender. Furthermore, all data were collected following rigorous methodology, including a study protocol with standardized quality control checks. The main limitation of our study is the cross-sectional nature of NHANES, which precludes conclusions regarding the temporal nature of the association between serum uric acid levels and diabetes mellitus. Therefore, even though we are able to detect an association that is statistically independent of traditional confounding factors such as age, BMI, and serum cholesterol, we are not able to establish which one is the cause and which the is an effect. Prospective studies are needed to establish the time sequence in the relationship between uric acid and diabetes mellitus. In summary, in a multiethnic sample of US adults, we found that higher serum uric acid levels are inversely associated with diabetes mellitus in both men and women.

##  Conflict of Interests

There are no conflict of interests related to this paper.

##  Disclosure

The guarantor, A. Shankar, accepts full responsibility for the work and/or the conduct of the study, had access to the data, and controlled the decision to publish.

##  Authors' Contributions

All the authors contributed to the intellectual development of this paper. P. Bandaru wrote the first draft of the paper. A. Shankar had the original idea for the study, was involved in critical revisions to the paper and is the guarantor.

## Figures and Tables

**Table 1:** Characteristics of the study population by categories of serum uric acid level^∗†^.

Characteristics	Quartile 1	Quartile 2	Quartile 3	Quartile 4	*P* value
Age, years	43.91 ± 0.31	43.66 ± 0.31	46.58 ± 0.31	50.71 ± 0.31	<0.0001
Women, %	53.52 ± 1.57	55.77 ± 1.18	49.49 ± 1.26	51.01 ± 1.12	0.0073
Race-ethnicity, %					0.0010
Non-Hispanic whites	77.75 ± 1.65	78.17 ± 1.45	75.53 ± 1.43	75.59 ± 1.53	
Non-Hispanic blacks	9.73 ± 0.67	9.84 ± 0.65	9.71 ± 0.73	12.45 ± 0.88	
Mexican Americans	5.60 ± 0.49	5.17 ± 0.42	5.32 ± 0.58	4.62 ± 0.42	
Others	6.92 ± 1.17	6.82 ± 1.04	9.44 ± 1.15	7.35 ± 0.89	
Education categories, %					0.0002
Below high school	22.36 ± 1.40	21.59 ± 1.14	22.71 ± 1.33	27.19 ± 1.35	
High school	32.19 ± 1.35	35.88 ± 1.19	33.47 ± 1.52	34.56 ± 0.99	
Above high school	45.45 ± 2.11	42.53 ± 1.63	43.82 ± 1.66	38.25 ± 1.38	
Smoking, %					<0.0001
Never smoker	49.08 ± 1.45	46.21 ± 1.52	50.17 ± 1.64	47.05 ± 1.31	
Former smoker	20.21 ± 1.07	22.78 ± 1.02	23.91 ± 1.19	28.22 ± 0.98	
Current smoker	30.72 ± 1.21	31.01 ± 1.62	25.92 ± 1.33	24.74 ± 1.18	
Alcohol intake, %					
Current drinker	54.36 ± 1.99	55.17 ± 1.80	57.91 ± 1.45	53.44 ± 1.81	0.0680
Body mass index, kg/m^2^					<0.0001
Normal	63.90 ± 1.40	53.22 ± 1.14	38.79 ± 1.28	24.36 ± 0.88	
Overweight	26.95 ± 1.10	32.28 ± 0.89	36.33 ± 1.44	35.97 ± 1.31	
Obese	9.16 ± 0.77	14.50 ± 0.98	24.88 ± 1.11	39.67 ± 1.22	
Total cholesterol, mg/dL	195.03 ± 0.73	200.54 ± 0.72	207.12 ± 0.73	214.81 ± 0.73	<0.0001

*Data presented are row percentages or mean values ± standard error (SE).

^†^Serum uric acid quartiles: <4.3 mg/dL, 4.30–5.20 mg/dL, 5.30–6.20 mg/dL, >6.20 mg/dL.

**Table 2:** Association between serum uric acid level and diabetes mellitus.

Serum uric acid level*	Number at risk (Diabetes cases)	Age- and sex-adjusted odds ratio (95% confidence interval)	Multivariable-adjusted odds ratio (95% confidence interval)^†^
Quartile 1 (<4.30 mg/dL)	3482 (305)	1 (referent)	1 (referent)
Quartile 2 (4.30–5.20 mg/dL)	3624 (206)	0.63 (0.44–0.91)	0.54 (0.36–0.80)
Quartile 3 (5.30–6.20 mg/dL)	3499 (185)	0.62 (0.45–0.85)	0.40 (0.29–0.56)
Quartile 4 (>6.20 mg/dL)	3539 (325)	0.54 (0.30–097)	0.48 (0.35–0.66)
*P* trend		0.0023	<0.0001

*Serum uric acid quartiles: <4.3 mg/dL, 4.30–5.20 mg/dL, 5.30–6.20 mg/dL, >6.20 mg/dL.

^†^Adjusted for age (years), sex (men, women), race-ethnicity (non-Hispanic whites, non-Hispanic blacks, Mexican Americans, etc.), education categories (<high school, high school, >high school), smoking (never, former, current), alcohol intake (never, former, current), BMI (normal, overweight, obese), hypertension (absent, present), and serum total cholesterol (mg/dL).

**Table 3:** Association between serum uric acid level and diabetes mellitus by gender.

Serum uric acid level*	Number at risk (Diabetes cases)	Multivariable-adjusted odds ratio (95% confidence interval)^†^
*Women*		
Quartile 1 (<3.8 mg/dL)	1839 (113)	1 (referent)
Quartile 2 (3.8–4.5 mg/dL)	2046 (121)	0.79 (0.49–1.28)
Quartile 3 (4.6–5.4 mg/dL)	1843 (103)	0.53 (0.33–0.83)
Quartile 4 (>5.4 mg/dL)	1881 (209)	0.78 (0.51–1.20)
*P* trend		0.3590
*Men*		
Quartile 1 (<5.20 mg/dL)	1643 (192)	1 (referent)
Quartile 2 (5.20–5.9 mg/dL)	1578 (85)	0.42 (0.23–0.76)
Quartile 3 (6.0–6.8 mg/dL)	1656 (82)	0.35 (0.21–0.58)
Quartile 4 (>6.8 mg/dL)	1658 (116)	0.32 (0.20–0.51)
*P* trend		<0.0001

*Serum uric acid quartiles: <4.3 mg/dL, 4.30–5.20 mg/dL, 5.30–6.20 mg/dL, >6.20 mg/dL.

^†^Adjusted for age (years), race-ethnicity (non-Hispanic whites, non-Hispanic blacks, Mexican Americans, etc.), education categories (<high school, high school, >high school), smoking (never, former, current), alcohol intake (never, former, current), BMI (normal, overweight, obese), hypertension (absent, present), and serum total cholesterol (mg/dL).

**Table 4:** Association between serum uric acid level and diabetes mellitus by hypertension.

Serum uric acid level*	Number at risk (Diabetes cases)	Multivariable-adjusted odds ratio (95% confidence interval)^†^
*No hypertension*		
Quartile 1 (<4.30 mg/dL)	2623 (150)	1 (referent)
Quartile 2 (4.30–5.20 mg/dL)	2651 (89)	0.45 (0.25–0.80)
Quartile 3 (5.30–6.20 mg/dL)	2271 (54)	0.29 (0.16–0.54)
Quartile 4 (>6.20 mg/dL)	1721 (77)	0.40 (0.22–0.70)
*P* trend		0.0015
*Hypertension*		
Quartile 1 (<4.30 mg/dL)	859 (155)	1 (referent)
Quartile 2 (4.30–5.20 mg/dL)	973 (117)	0.66 (0.43–1.01)
Quartile 3 (5.30–6.20 mg/dL)	1228 (131)	0.52 (0.35–0.76)
Quartile 4 (>6.20 mg/dL)	1818 (248)	0.57 (0.38–0.86)
*P* trend		0.0232

*Serum uric acid quartiles: <4.3 mg/dL, 4.30–5.20 mg/dL, 5.30–6.20 mg/dL, >6.20 mg/dL.

^†^Adjusted for age (years), sex (men, women), race-ethnicity (non-Hispanic whites, non-Hispanic blacks, Mexican Americans, etc.), education categories (<high school, high school, >high school), smoking (never, former, current), alcohol intake (never, former, current), BMI (normal, overweight, obese), and serum total cholesterol (mg/dL).
